# Generative Embedding for Model-Based Classification of fMRI Data

**DOI:** 10.1371/journal.pcbi.1002079

**Published:** 2011-06-23

**Authors:** Kay H. Brodersen, Thomas M. Schofield, Alexander P. Leff, Cheng Soon Ong, Ekaterina I. Lomakina, Joachim M. Buhmann, Klaas E. Stephan

**Affiliations:** 1 Department of Computer Science, ETH Zurich, Zurich, Switzerland; 2 Laboratory for Social and Neural Systems Research, Department of Economics, University of Zurich, Zurich, Switzerland; 3 Wellcome Trust Centre for Neuroimaging, University College London, London, United Kingdom; Indiana University, United States of America

## Abstract

Decoding models, such as those underlying multivariate classification algorithms, have been increasingly used to infer cognitive or clinical brain states from measures of brain activity obtained by functional magnetic resonance imaging (fMRI). The practicality of current classifiers, however, is restricted by two major challenges. First, due to the high data dimensionality and low sample size, algorithms struggle to separate informative from uninformative features, resulting in poor generalization performance. Second, popular discriminative methods such as support vector machines (SVMs) rarely afford mechanistic interpretability. In this paper, we address these issues by proposing a novel generative-embedding approach that incorporates neurobiologically interpretable generative models into discriminative classifiers. Our approach extends previous work on trial-by-trial classification for electrophysiological recordings to subject-by-subject classification for fMRI and offers two key advantages over conventional methods: it may provide more accurate predictions by exploiting discriminative information encoded in ‘hidden’ physiological quantities such as synaptic connection strengths; and it affords mechanistic interpretability of clinical classifications. Here, we introduce generative embedding for fMRI using a combination of dynamic causal models (DCMs) and SVMs. We propose a general procedure of DCM-based generative embedding for subject-wise classification, provide a concrete implementation, and suggest good-practice guidelines for unbiased application of generative embedding in the context of fMRI. We illustrate the utility of our approach by a clinical example in which we classify moderately aphasic patients and healthy controls using a DCM of thalamo-temporal regions during speech processing. Generative embedding achieves a near-perfect balanced classification accuracy of 98% and significantly outperforms conventional activation-based and correlation-based methods. This example demonstrates how disease states can be detected with very high accuracy and, at the same time, be interpreted mechanistically in terms of abnormalities in connectivity. We envisage that future applications of generative embedding may provide crucial advances in dissecting spectrum disorders into physiologically more well-defined subgroups.

## Author Summary

Neurological and psychiatric spectrum disorders are typically defined in terms of particular symptom sets, despite increasing evidence that the same symptom may be caused by very different pathologies. Pathophysiological classification and effective treatment of such disorders will increasingly require a mechanistic understanding of inter-individual differences and clinical tools for making accurate diagnostic inference in individual patients. Previous classification studies have shown that functional magnetic resonance imaging (fMRI) can be used to differentiate between healthy controls and neurological or psychiatric patients. However, these studies are typically based on descriptive patterns and indirect measures of neural activity, and they rarely afford mechanistic insights into the underlying condition. In this paper, we address this challenge by proposing a classification approach that rests on a model of brain function and exploits the rich discriminative information encoded in directed interregional connection strengths. Based on an fMRI dataset acquired from moderately aphasic patients and healthy controls, we illustrate that our approach enables more accurate classification and deeper mechanistic insights about disease processes than conventional classification methods.

## Introduction

Recent years have seen a substantial increase in the use of functional neuroimaging data for investigating healthy brain function and detecting abnormalities. The most popular type of analysis is statistical parametric mapping (SPM), a mass-univariate encoding model of fMRI data in which the statistical relationship between experimental (or clinical) variables and haemodynamic measurements of neural activity is examined independently for every voxel in the brain [1]. While this approach has led to many insights about functional abnormalities in psychiatric and neurological disorders, it suffers from two limitations. First, since univariate models are insensitive to spatially distributed patterns of neural activity, they may fail to detect subtle, distributed differences between patients and healthy controls that are not expressed as local peaks or clusters of activity [2]. Second, while encoding models such as SPM are excellent for describing regional differences in brain activity across clinical groups, they are less well suited for clinical decision making, where the challenge is to predict the disease state of an individual subject from measured brain activity [3]–[5].

An alternative approach is provided by multivariate decoding methods, in particular classification algorithms. Unlike mass-univariate encoding models, these methods predict an experimental variable (e.g., a trial-specific condition or subject-specific disease state) from the activity pattern across voxels (see [6]–[10] for reviews). Using multivariate decoding models instead of mass-univariate encoding models has interesting potential for clinical practice, particularly for diseases that are difficult to diagnose. Consequently, much work is currently being invested in constructing classifiers that can predict the diagnosis of individual subjects from structural or functional brain data [11], [3], [12], [13], [4], [14]–[16]. Historically, these efforts date back to positron emission tomography (PET) studies in the early 1990s [8]. Today, attempts of using multivariate classifiers for subject-by-subject diagnosis largely focus on MRI and fMRI data [11], [3], [12], [17].

### Challenges for current classification approaches

Despite their increasing popularity, two challenges critically limit the practical applicability of current classification methods for functional neuroimaging data. First, classifying subjects directly in voxel space is often a prohibitively difficult task. This is because functional neuroimaging datasets (i) typically exhibit a low signal-to-noise ratio, (ii) are obtained in an extremely high-dimensional measurement space (a conventional fMRI scan contains more than 100,000 voxels), and (iii) are characterized by a striking mismatch between the large number of voxels and the small number of available subjects. As a result, even the most carefully designed algorithms have great difficulties in reliably finding jointly informative voxels while ignoring uninformative sources of noise. Popular strategies include: preselecting voxels based on an anatomical mask [18], or a separate functional localizer [20], [21]; spatial subsampling [22]; finding informative voxels using univariate models [3], [11], [12] or locally multivariate searchlight methods [23], [24]; and unsupervised dimensionality reduction [4], [25]. Other recently proposed strategies attempt to account for the inherent spatial structure of the feature space [23], [26], [27] or use voxel-wise models to infer a particular stimulus identity [28]–[30]. Finally, those submissions that performed best in the Pittsburgh Brain Activity Interpretation Competition (PBAIC 2007) highlighted the utility of kernel ridge regression [31] and relevance vector regression [31], [32]. The common assumption underlying all of these approaches is that interesting variations of the data with regard to the class variable are confined to a manifold that populates a latent space of much lower dimensionality than the measurement space.

The second challenge for classification methods concerns the interpretation of their results. Most classification studies to date draw conclusions from overall prediction accuracies [33], [11], the spatial deployment of informative voxels [19], [34], [18], [35]–[39], the temporal evolution of discriminative information [40], [37], [41], [42], [26], or patterns of undirected regional correlations [43]. These approaches may support discriminative decisions, but they are blind to the neuronal mechanisms (such as effective connectivity or synaptic plasticity) that underlie discriminability of brain or disease states. In other words: while some conventional classification studies have achieved impressive diagnostic accuracy [14], their results have not improved our mechanistic understanding of disease processes.

### Generative embedding

**Figure 1:**
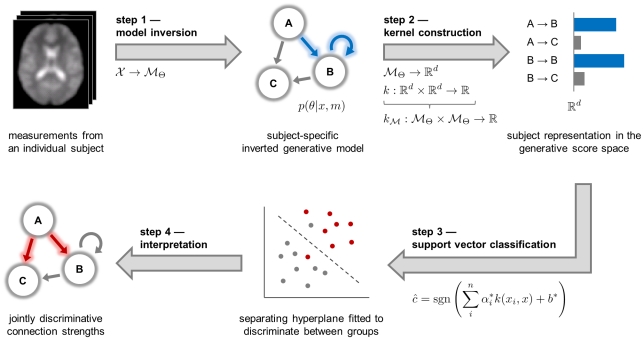
Conceptual overview of generative embedding for fMRI. This schematic illustrates the key principles by which generative embedding enables model-based classification for functional magnetic resonance imaging (fMRI). Initially, each subject is represented by a measure of blood oxygen level dependent (BOLD) activity with one temporal and three spatial dimensions. In the first analysis step (model inversion), these subject-specific data are used to estimate the parameters of a generative model, which represents a mapping of the data 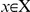 onto a probability distribution 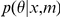 in a parametric family 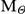 (see Sections ‘DCM for fMRI’ and ‘Model inversion’). In the second step (kernel construction), a kernel function 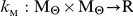 is defined that represents a similarity metric between any two fitted models 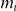 and 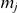. This step can be split up into an initial mapping 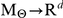 followed by a conventional kernel 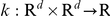. The kernel implies a generative score space (or model-based feature space; see Section ‘Kernel construction’), which provides a comprehensive statistical representation of every subject. In this illustrative participant, the influence of region A on region B as well as the self-connection of region B were particularly strong. In the third step, a classifier is used to find a separating hyperplane between groups of subjects, based exclusively on their model-based representations (see Section ‘Classification’). When using a linear kernel, each feature corresponds to the coupling strength between two regions, which, in the fourth step, enables a mechanistic interpretation of feature weights in the context of the underlying model (see Section ‘Interpretation of the feature space’). Here, the influence of A on B and C were jointly most informative in distinguishing between groups. For a concrete implementation of this procedure, see Figure 2.

Generative embedding for model-based classification may provide a solution to the challenges outlined above. It is based on the idea that both the performance and interpretability of conventional approaches could be improved by taking into account available prior knowledge about the process generating the observed data (see [44] for an overview). (The term *generative embedding* is sometimes used to denote a particular model-induced feature space, or so-called generative score space, in which case the associated line of research is said to be concerned with *generative embeddings*. Here, we will use the term in singular form to denote the process of using a generative model to project the data into a generative score space, rather than using the term to denote the space itself.) Generative embedding rests on two components: a generative model for principled selection of mechanistically interpretable features and a discriminative method for classification (see Figure 1).

*Generative models* have proven powerful in explaining how observed data are caused by the underlying (neuronal) system. Unlike their discriminative counterparts, generative models capture the joint probability of the observed data and the class labels, governed by a set of parameters of a postulated generative process. One example in neuroimaging is *dynamic causal modelling* (DCM) [45]. DCM enables statistical inference on physiological quantities that are not directly observable with current methods, such as directed interregional coupling strengths and their modulation, e.g., by synaptic gating [46]. (We use the term *DCM* to refer both to a specific dynamic causal model and to dynamic causal modelling as a method.) From a pathophysiological perspective, disturbances of synaptic plasticity and neuromodulation are at the heart of psychiatric spectrum diseases such as schizophrenia [47] or depression [48]. It is therefore likely that classification of disease states could benefit from exploiting estimates of these quantities. While DCM is a natural (and presently the only) candidate for obtaining model-based estimates of synaptic plasticity (cf. [46], [49]), the most widely used approach to classification relies on *discriminative methods*, such as support vector machines (SVMs) [50], [51]. Together, DCM and SVM methods thus represent natural building blocks for classification of disease states.

Generative embedding represents a special case of using *generative kernels* for classification, such as the *P*-kernel [52] or the Fisher kernel [53]. Generative kernels have been fruitfully exploited in a range of applications [54]–[66] and define an active area of research [67]–[70]. In the special case of generative embedding, a generative kernel is used to construct a *generative score space*. This is a model-based feature space in which the original observations have been replaced by statistical representations that potentially yield better class separability when fed into a discriminative classifier. Thus, an unsupervised embedding step is followed by a supervised classification step. In previous work, we suggested a concrete implementation of this approach for the trial-by-trial classification of electrophysiological recordings [61]. In this paper, we propose a DCM-based generative-embedding approach for subject-by-subject classification of fMRI data, demonstrate its performance using a clinical data set, and highlight potential methodological pitfalls (and how to avoid them).

DCM [45] views the brain as a nonlinear dynamical system of interconnected neuronal populations whose directed connection strengths are modulated by external perturbations (i.e., experimental conditions) or endogenous activity. Here, we will use DCM to replace high-dimensional fMRI time series by a low-dimensional vector of parameter estimates. The discriminative part of our approach will be based on an SVM with a linear kernel. This algorithm learns to discriminate between two groups of subjects by estimating a separating hyperplane in their feature space. Since this paper brings together techniques from different statistical domains that tend to be used by different communities, we have tried to adopt a tutorial-like style and introduce basic concepts of either approach in the Methods section.

Generative embedding for fMRI may offer three substantial advantages over conventional classification methods. First, because the approach aims to fuse the strengths of generative models with those of discriminative methods, it may outperform conventional voxel-based schemes, especially in those cases where crucial discriminative information is encoded in ‘hidden’ quantities such as directed (synaptic) connection strengths. Second, the construction of the feature space is governed and constrained by a biologically motivated systems model. As a result, feature weights can be interpreted mechanistically in the context of this model. Incidentally, the curse of dimensionality faced by many conventional feature-extraction methods may turn into a blessing when using generative embedding: the higher the temporal and spatial resolution of the fMRI data, the more precise the estimation of the parameters of the generative model, leading to better discriminability. Third, our approach can be used to compare alternative generative model architectures in situations where evidence-based approaches, such as Bayesian model selection, are not applicable. We will deal with these three points in more detail in the Discussion.

### Structure of this paper

The remainder of this paper is structured as follows. First, we summarize the general ideas of generative embedding and the specific generative and discriminative components used here, i.e., DCM and SVM. We then inspect different procedures of how generative embedding could be implemented practically while distinguishing between approaches with and without bias. Third, we illustrate the utility of our approach, using empirical data obtained during speech processing in healthy volunteers and patients with moderate aphasia. These data have been explored in a previous study, in which DCM and Bayesian model selection (BMS) were applied to investigate the effective connectivity among cortical areas activated by intelligible speech [71]. In a subsequent study, we extended this analysis to patients with aphasia (Schofield et al., *in preparation*). In the present paper, we ask whether subject-specific directed connection strengths among cortical regions involved in speech processing contain sufficiently rich discriminative information to enable accurate predictions of the diagnostic category (healthy or aphasic) of a previously unseen individual. In brief, we found that (i) generative embedding yielded a near-perfect classification accuracy, (ii) significantly outperformed conventional ‘gold standard’ activation-based and correlation-based classification schemes, and (iii) afforded a novel mechanistic interpretation of the differences between aphasic patients and healthy controls during processing of speech and speech-like sounds.

## Methods

### Ethics statement

The study was approved by the local research ethics committee at UCL, and all participants gave informed consent.

### Combining generative models and discriminative methods

Most methods for classification attempt to find a linear function that separates examples as accurately as possible in a space of features (e.g., voxel-wise measurements). Such *discriminative* classification methods differ from *generative* methods in two ways. First, rather than trying to estimate the joint density of observations and class labels, which is not needed for classification, or trying to estimate class-conditional probability densities, which can be difficult, discriminative classifiers directly model the class an example belongs to. Second, many discriminative methods do not operate on examples themselves but are based on the similarity between any two examples, expressed as the inner product between their feature vectors. This provides an elegant way of transforming a linear classifier into a more powerful nonlinear one. (Note that the term *discriminative methods* is used here to collectively describe the class of learning algorithms that find a *discriminant function* for mapping an example 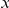 onto a class label 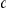, typically without invoking probability theory. This is in contrast to *discriminative models,* which model the conditional probability 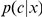, and *generative models,* which first model the full joint probability 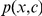 and then derive 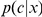.)

The most popular classification algorithm of the above kind is the 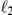-norm soft-margin support vector machine (SVM) [50], [51], [72], [73]. The only way in which examples 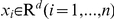 enter an SVM is in terms of an inner product 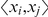. This product can be replaced by the evaluation 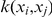 of a *kernel function* 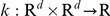, which implicitly computes the inner product between the examples in a new feature space, 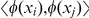.

The 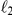-norm SVM is a natural choice when the goal is maximal prediction accuracy. However, it usually leads to a dense solution (as opposed to a sparse solution) in which almost all features are used for classification. This is suboptimal when one wishes to understand which model parameters contribute most to distinguishing groups, which will be the focus in the Section ‘Interpretation of the feature space.’ In this case, an SVM that enforces *feature sparsity* may be more useful. One simple way of inducing sparsity is to penalize the number of non-zero coefficients by using an 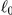-regularizer. Unlike other regularizers, the 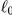-norm (also known as the *counting norm*) reduces the feature-selection bias inherent in unbounded regularizers such as the 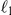
- or 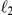-norm. The computational cost of optimizing an 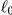-SVM objective function is prohibitive, because the number of subsets of 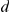 items which are of size 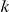 is exponential in 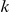. We therefore replace the 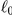-norm by a capped 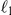-regularizer which has very similar properties [74]. One way of solving the resulting optimization problem is to use a bilinear programming approach [75]. Here, we use a more efficient difference-of-convex-functions algorithm (Ong & Thi, *under review*).

In summary, we will use two types of SVM. For the purpose of classification (Section ‘Classification’), we aim to maximize the potential for highly accurate predictions by using an 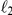-norm SVM. For the purpose of feature selection and interpretation (Section ‘Interpretation of the feature space’), we will focus on feature sparsity by using an approximation to an 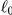-norm SVM, which will highlight those DCM parameters jointly deemed most informative in distinguishing between groups.

Most current applications of classification algorithms in neuroimaging begin by embedding the measured recordings of each subject in a 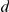-dimensional Euclidean space 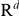. In fMRI, for example, a subject can be represented by a vector of 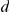 features, each of which corresponds to the signal measured in a particular voxel at a particular point in time. This approach makes it possible to use any learning algorithm that expects vectorial input, such as an SVM; but it ignores the spatio-temporal structure of the data as well as the process that generated them. This limitation has motivated the search for kernel methods that provide a more natural way of measuring the similarity between the functional datasets of two subjects, for example by incorporating prior knowledge about how the data were generated, which has led to the idea of *generative kernels*, as described below.

Generative kernels are functions that define a similarity metric for observed examples using a generative model. In the case of a dynamic causal model (DCM), for example, the observed time series are modelled by a system of parameterized differential equations with Gaussian observation noise. Generative embedding defines a generative kernel by transferring the models into a vectorial feature space in which an appropriate similarity metric is defined (see Figure 1). This feature space, which we will refer to as a *generative score space,* embodies a model-guided dimensionality reduction of the observed data. The kernel defined in this space could be a simple inner product of feature vectors, or it could be based on any other higher-order function, as long as it is positive definite [76]. In conclusion, model-based classification via generative embedding is a hybrid generative-discriminative approach: it merges the explanatory abilities of generative models with the classification power of discriminative methods.

The specific implementation for fMRI data proposed in this paper consists of four conceptual steps which are summarized in Figure 1 and described in the following subsections. First, a mapping 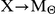 is designed that projects an example 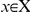 from data space onto a multivariate probability distribution in a parametric family 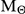. In our case, we use the fMRI data from each subject to estimate the posterior density of the parameters of a DCM (Sections ‘DCM for fMRI’ and ‘Model inversion’). Second, a probability kernel 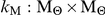 is constructed that represents a similarity measure between two inverted DCMs. Here, we use a simple linear kernel on the *maximum a posteriori* (MAP) estimates of the model parameters (Sections ‘Strategies for unbiased model specification and inversion’ and ‘Kernel construction’). Third, this kernel is used for training and testing a discriminative classifier (Section ‘Classification’). Here, we employ a linear SVM to distinguish between patients and healthy controls. Fourth, the constructed feature space can be investigated to find out which model parameters jointly contributed most to distinguishing the two groups (Section ‘Interpretation of the feature space’). We will conclude with an example in which we distinguish between patients with moderate aphasia and healthy controls (Sections ‘Experimental design, data acquisition, and preprocessing,’ ‘Implementation of generative embedding,’ and ‘Comparative analyses’).

### DCM for fMRI

DCM regards the brain as a nonlinear dynamic system of interconnected nodes, and an experiment as a designed perturbation of the system's dynamics [45]. Its goal is to provide a mechanistic model for explaining experimental measures of brain activity. While the mathematical formulation of DCMs varies across measurement types, common mechanisms modelled by all DCMs include synaptic connection strengths and experimentally induced modulation thereof [46], [77]–[80]. Generally, DCMs strive for neurobiological interpretability of their parameters; this is one core feature distinguishing them from alternative approaches, such as multivariate autoregressive models [81] which characterize inter-regional connectivity in a phenomenological fashion.

DCMs consist of two hierarchical layers [82]. The first layer is a *neuronal model* of the dynamics of interacting neuronal populations in the context of experimental perturbations. Critically, its parameters are neurobiologically interpretable, representing, for example, synaptic weights and their context-specific modulation; electrophysiological DCMs describe even more fine-grained processes such as spike-frequency adaptation or conduction delays. Experimental manipulations 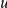 enter the model in two different ways: they can elicit responses through direct influences on specific regions (e.g., sensory inputs), or they can modulate the strength of coupling among regions (e.g., task demands or learning). The second layer of a DCM is a biophysically motivated *forward model* that describes how a given neuronal state translates into a measurement. Depending on the measurement modality, this can be a set of nonlinear differential equations (as for fMRI [83]) or a simple linear equation (as for EEG [84]). While the forward model plays a critical role in model inversion, it is the parameters of the neuronal model that are typically of primary scientific interest.

In this paper, we will use the classical bilinear DCM for fMRI [45] as implemented in the software package SPM8/DCM10,
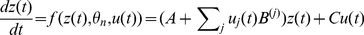
(1)

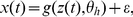
(2)where 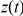 represents the neuronal state vector 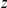 at time 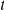, 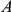 is a matrix of endogenous connection strengths, 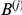 represents the additive change of these connection strengths induced by modulatory input 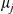, and 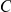 denotes the strengths of direct (driving) inputs. These neuronal parameters 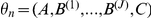 are rate constants with units 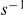.

The haemodynamic forward model is given by the function 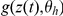, a nonlinear operator that links a neuronal state 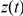 to a predicted blood oxygen level dependent (BOLD) signal via changes in vasodilation, blood flow, blood volume, and deoxyhaemoglobin content (see [83] for details). This forward model has haemodynamic parameters 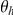 and Gaussian measurement error 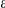. The haemodynamic parameters primarily serve to account for variations in neurovascular coupling across regions and subjects and are typically not of primary scientific interest. In addition, the haemodynamic parameters exhibit strong inter-dependencies and thus high posterior covariances and low precision [83], which makes it difficult to establish the distinct contribution afforded by each parameter. For these reasons, the model-induced feature spaces in this paper will be based exclusively on the neuronal parameters 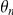.

In summary, DCM provides a mechanistic model for explaining measured time series of brain activity as the outcome of hidden dynamics in an interconnected network of neuronal populations and its experimentally induced perturbations. Inverting such a model (see next section) means to infer the posterior distribution of the parameters of both the neuronal and the forward model from observed responses of a specific subject. Its mechanistic interpretability and applicability to single-subject data makes DCM an attractive candidate for generative embedding of fMRI data.

### Model inversion

Bayesian inversion of a given dynamic causal model 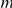 defines a map 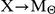 that projects a given example 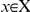 (i.e., data from a single subject) onto a multivariate probability distribution 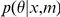 in a parametric family 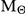. The model architecture 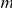 specifies the neuronal populations (regions) of interest, experimentally controlled inputs 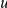, synaptic connections, and a prior distribution over the parameters 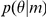. Given the model 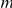 and subject-specific data 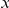, model inversion proceeds in an unsupervised and subject-by-subject fashion, i.e., in ignorance of the subject label that will later be used in the context of classification. (The literature on DCM has adopted the convention of denoting the hidden states by 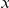 and the data by 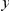. Here, in order to keep the notation consistent with the literature on classification, we use 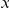 for the data and 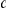 for the labels. A distinct symbol for the hidden states is not required here.) DCM uses a fully Bayesian approach to parameter estimation, with empirical priors for the haemodynamic parameters and conservative shrinkage priors for the coupling parameters [85], [45]. Combining the prior density over the parameters 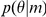 with the likelihood function 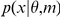 yields the posterior density 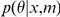. This inversion can be carried out efficiently by maximizing a variational free-energy bound to the log model evidence, 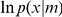, under Gaussian assumptions about the posterior (the Laplace assumption; see [86] for details). Given 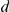 parameters, model inversion thus yields a subject-specific probability density 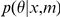 that can be fully described in terms of a vector of posterior means 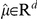 and a covariance matrix 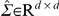.

Model specification and selection is an important theme in DCM [87]. In this paper we are not concerned with the question of which of several alternative DCMs may be optimal for explaining the data or for classifying subjects; these issues can be addressed using Bayesian evidence methods [88], [89] or by applying cross-validation to the classifications suggested by each of the models, respectively (see [61] for an example). However, an important issue is that model specification cannot be treated in isolation from its subsequent use for classification. Specifically, some procedures for selecting time series can lead to biased estimation of classification accuracy. In the next section, we therefore provide a detailed assessment of different strategies for time series selection in DCM-based generative embedding and highlight those procedures which safeguard against obtaining optimistic estimates of classification performance.

### Strategies for unbiased model specification and inversion

**Figure 2:**
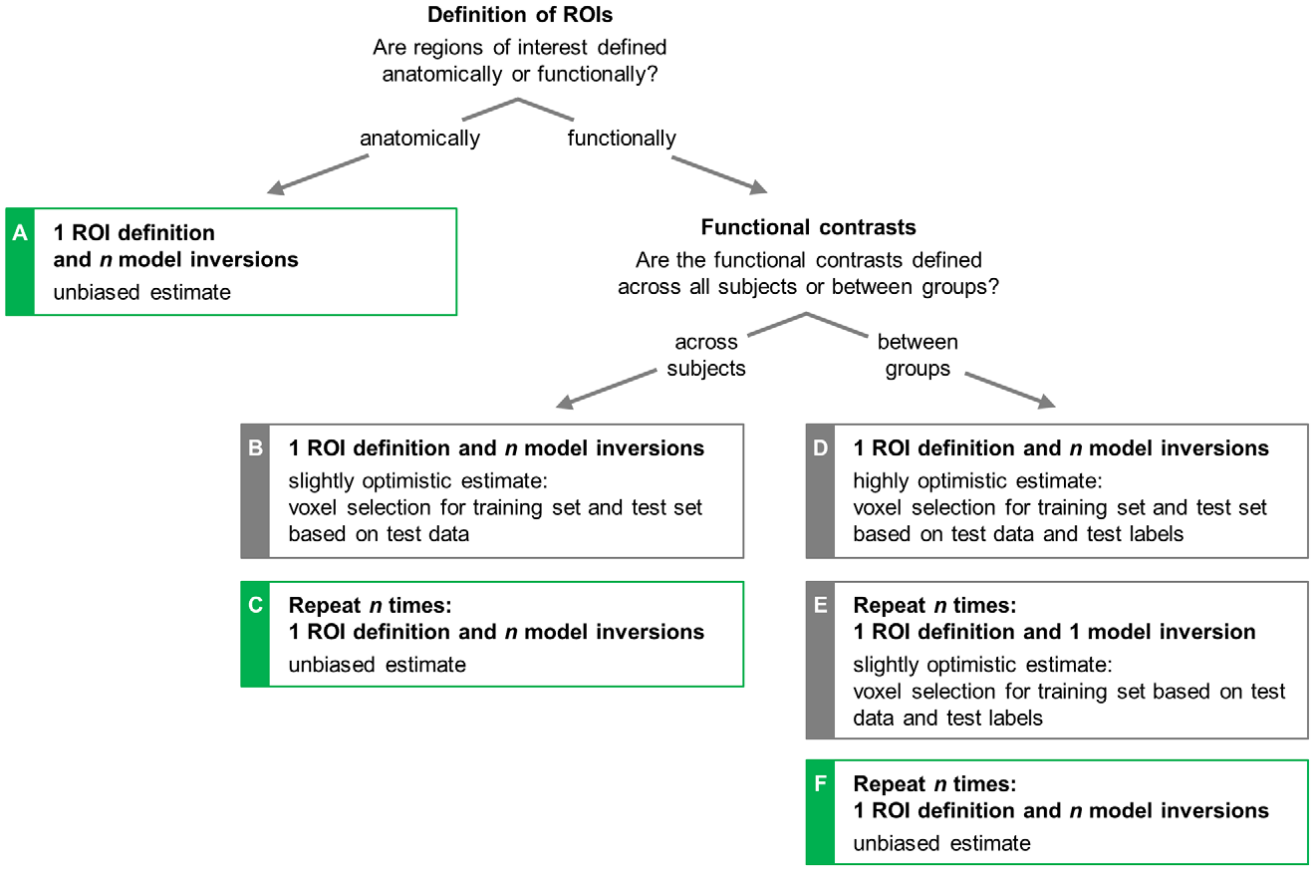
Strategies for unbiased DCM-based generative embedding. This figure illustrates how generative embedding can be implemented using dynamic causal modelling. Depending on whether regions of interest are defined anatomically, based on across-subjects functional contrasts, or based on between-group contrasts, there are several possible practical procedures. Some of these procedures may lead to biased estimates of classification accuracy (grey boxes). Procedures *a*, *c*, and *f* avoid this bias, and are therefore recommended (green boxes). The analysis of the illustrative dataset described in this paper follows procedure *c*.

For conventional fMRI classification procedures, good-practice guidelines have been suggested for avoiding an optimistic bias in assessing classification performance [8], [10]. Generally, to obtain an unbiased estimate of generalization accuracy, a classifier must be applied to test data that have not been used during training. In generative embedding, this principle implies that the specification of the generative model cannot be treated in isolation from its use for classification. In this section, we structure different strategies in terms of a decision tree and evaluate the degree of bias they invoke (see Figure 2).

The first distinction is based on whether the regions of interest (ROIs) underlying the DCM are defined *anatomically* or *functionally*. When ROIs are defined exclusively on the basis of anatomical masks (Figure 2a), the selection of voxels is independent of the functional data. Using time series from these regions, the model is inverted separately for each subject. Thus, given 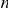 subjects, a single initial model-specification step is followed by 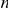 subject-wise model inversions. The resulting parameter estimates can be safely submitted to a cross-validation procedure to obtain an unbiased estimate of classification performance.

Whenever *functional* contrasts have played a role in defining ROIs, subsequent classification may no longer be unbiased. This is because a functional contrast introduces statistics of the data into voxel selection, which usually generates a bias. In this case, we ask whether contrasts are defined in an *across-subjects* or a *between-groups* fashion. In the case of an across-subjects contrast (which does not take into account group membership), one might be tempted to follow the same logic as in the case of anatomical ROI definitions: a single across-subjects contrast, computed for all subjects, guides the selection of voxels, and the resulting DCM is inverted separately for each subject (Figure 2b). Unfortunately, this procedure is problematic. When using the resulting parameter estimates in a leave-one-out cross-validation scheme, in every repetition the features would be based on a model with regions determined by a group contrast that was based on the data from all subjects, including the left-out test subject. This means that training the classifier would no longer be independent of the test data, which violates the independence assumption underlying cross-validation, a situation referred to as *peeking* [10]. In consequence, the resulting generalization estimate may exhibit an optimistic bias. To avoid this bias, model specification must be integrated into cross-validation (Figure 2c). Specifically, in each fold, we leave out one subject as a test subject and compute an across-subjects group contrast from the remaining 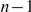 subjects. The resulting choice of voxels is then used for specifying time series in each subject and the resulting model is inverted separately for each subject, including the left-out test subject. This procedure is repeated 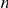 times, each time leaving out a different subject. In total, the model will be inverted 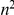 times. In this way, within each cross-validation fold, the selection of voxels is exclusively based on the training data, and no peeking is involved. This is the strategy adopted for the dataset analysed in this paper, as detailed in the Section ‘Implementation of generative embedding’.

When functional contrasts are not defined *across* all subjects but *between* groups, the effect of peeking may become particularly severe. Using a between-groups contrast to define regions of interest on the basis of all available data, and using these regions to invert the model for each subject (Figure 2d) would introduce information about group membership into the process of voxel selection. Thus, feature selection for both training and test data would be influenced by both the data and the label of the left-out test subject. One way of decreasing the resulting bias is to integrate model specification into cross-validation (Figure 2e). In this procedure, the between-groups contrast is computed separately for each training set (i.e., based on 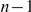 subjects), and the resulting regions are used to invert the model for the test subject. This means that the class label of the test subject is no longer involved in selecting features for the test subject. However, the test label continues to influence the features of the training set, since these are based on contrasts defined for a group that included the test subject. This bias can only be removed by adopting the same laborious procedure as with across-subjects contrasts: by using a between-groups contrast involving 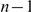 subjects, inverting the resulting model separately for each subject, and repeating this procedure 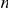 times (Figure 2f). This procedure guarantees that neither the training procedure nor the features selected for the test subject were influenced by the data or the label of the test subject.

In summary, the above analysis shows that there are three practical strategies for the implementation of generative embedding that yield an unbiased cross-validated accuracy estimate. If regions are defined anatomically, the model is inverted separately for each subject, and the resulting parameter estimates can be safely used in cross-validation (Figure 2a). Otherwise, if regions are defined by a functional contrast, both the definition of ROIs and model inversion for all subjects need to be carried out separately for each cross-validation fold (Figure 2c,f).

### Kernel construction

Given a set of inverted subject-specific generative models, the kernel defines the similarity metric under which these models are assessed within a discriminative classifier. In generative embedding, the choice of an appropriate kernel depends on the definition of the generative score space. A straightforward way to create a Euclidean vector space from an inverted DCM is to consider the posterior means or *maximum a posteriori* (MAP) estimates of model parameters of interest (e.g., parameters encoding synaptic connection strengths). More formally, we can define a mapping 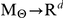 that extracts a subset of MAP estimates 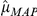 from the posterior distribution 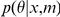. This simple 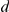-dimensional vector space expresses discriminative information encoded in the connection strengths between regions, as opposed to activity levels within these regions. Alternatively, one could also incorporate elements of the posterior covariance matrix into the vector space. This would be beneficial if class differences were revealed by the precision with which connection strengths can be estimated from the data.

Once a generative score space has been created, any conventional kernel 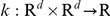 can be used to compare two inverted models. The simplest one is the linear kernel 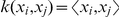, representing the inner product between two vectors 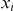 and 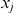. Nonlinear kernels, such as quadratic, polynomial or radial basis function kernels, transform the generative score space, which makes it possible to consider quadratic (or higher-order) class boundaries and therefore account for possible interactions between features. Nonlinear kernels, however, have several disadvantages for generative embedding. As the complexity of the kernel increases, so does the risk of overfitting. Furthermore, feature weights are easiest to interpret in relation to the underlying model when they do not undergo further transformation; then, the contribution of a particular feature (i.e., model parameter) to the success of the classifier can be understood as the degree to which the neuronal mechanism represented by that parameter aids classification. A simple linear kernel will therefore be our preferred choice.

In summary, in this paper, we define a mapping 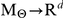 from a subject-specific posterior distribution of model parameters 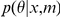 to a feature vector 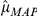. We then use a linear kernel 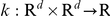 for this model-based feature space. Together, these two steps define a probability kernel 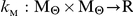 that represents a similarity metric between two inverted models and allows for mechanistic interpretations of how group membership of different subjects is encoded by spatiotemporal fMRI data.

### Classification

While a kernel describes how two subjects can be compared using a generative model of their fMRI data, it does not specify how such a comparison could be used for making predictions. This gap is filled by discriminative classification methods. As described in the Section ‘Combining generative models and discriminative methods’, a natural choice is the 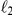-norm soft-margin support vector machine (SVM), which currently represents the most widely used kernel method for classification [72].

An estimate of classification performance with minimal variance can be obtained by leave-one-out cross-validation. In each fold, the classifier is trained on 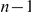 subjects and tested on the left-out one. Using the training set only, the SVM can be fine-tuned by carrying out a simple line search over the regularization hyperparameter 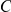 (Eqn. 1), a procedure known as nested cross-validation [90], [91].

There are many ways of assessing the generalization performance of a classifier. Here, we are primarily interested in the *balanced accuracy*, that is, the mean accuracy obtained on either class,
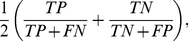
(3)where 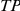, 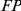, 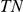, and 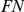 represent the number of true positives, false positives, true negatives, and false negatives, respectively [92]. The balanced accuracy represents the arithmetic mean between sensitivity and specificity. If the classifier performs equally well on either class, it reduces to the ordinary accuracy (i.e., the ratio of correct predictions to all predictions). If, however, the classifier has taken advantage of an imbalanced dataset, then the ordinary accuracy will be inflated, whereas the balanced accuracy will drop to chance (50%), as desired. The balanced accuracy thus removes the bias from estimates of generalizability that may arise in the presence of imbalanced datasets. A probability interval can be computed by considering the convolution of two Beta-distributed random variables that correspond to the true accuracies on positive and negative examples, respectively. A *p*-value can then be obtained by computing the posterior probability of the accuracy being below chance [92].

### Interpretation of the feature space

Most classification algorithms can not only be used for making predictions and obtaining an estimate of their generalization error; they can also be used to quantify how much each feature has contributed to classification performance. Such *feature weights* can sometimes be of greater interest than the classification accuracy itself. In the case of a generative score space, as defined above, each feature is associated with a neurobiologically interpretable model parameter. Provided there are no complex transformations of feature weights (see above), they can be interpreted in the context of the underlying model.

As described in the Section ‘Combining generative models and discriminative methods’, the 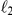-norm soft-margin SVM is a natural choice when the goal is maximal prediction accuracy. However, its solution usually implies that almost all features are used for classification. This is suboptimal when one wishes to understand which model parameters, and thus mechanisms, contribute most to distinguishing groups. Therefore, for the purposes of interpreting the model-induced feature space, we use an 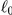-regularizer. This approach allows us to characterize the feature space by counting how often a particular feature has been selected in leave-one-out cross-validation.

### Experimental design, data acquisition, and preprocessing

In order to illustrate the utility of generative embedding for fMRI, we used data from two groups of participants (patients with moderate aphasia vs. healthy controls) engaged in a simple speech-processing task. The conventional SPM and DCM analyses of these data are published elsewhere; we refer to [71] and Schofield et al. (*in preparation*) for detailed descriptions of all experimental procedures.

The two groups of subjects consisted of 26 right-handed healthy participants with normal hearing, English as their first language, and no history of neurological disease (12 female; mean age 54.1 years; range 26–72 years); and 11 patients diagnosed with moderate aphasia due to stroke (1 female; mean age 66.1; range 45–90 years). The patients' aphasia profile was characterized using the Comprehensive Aphasia Test [93]. As a group, they had scores in the aphasic range for: spoken and written word comprehension (single word and sentence level), single word repetition and object naming. It is important to emphasize that the lesions did not affect any of the temporal regions which we included in our model described below (see Schofield et al., *in preparation*, for detailed information on lesion localization).

Subjects were presented with two types of auditory stimulus: (i) normal speech; and (ii) time-reversed speech, which is unintelligible but retains both speaker identity and the spectral complexity of normal speech. Subjects were given an incidental task, to make a gender judgment on each auditory stimulus, which they indicated with a button press.

Functional T2*-weighted echo-planar images (EPI) with BOLD contrast were acquired using a Siemens Sonata 1.5 T scanner (in-plane resolution 3 mm×3 mm; slice thickness 2 mm; inter-slice gap 1 mm; TR 3.15 s). In total, 122 volumes were recorded in each of 4 consecutive sessions. In addition, a T1-weighted anatomical image was acquired. Following realignment and unwarping of the functional images, the mean functional image of each subject was coregistered to its high-resolution structural image. This image was spatially normalized to standard Montreal Neurological Institute (MNI152) space, and the resulting deformation field was applied to the functional data. These data were then spatially smoothed using an isotropic Gaussian kernel (FWHM 8 mm). In previous work, these data have been analysed using a conventional general linear model (GLM) and DCM; the results are described in Schofield et al. (*in preparation*). Here, we re-examined the dataset using the procedure shown in Figure 2c, as described in detail in the next subsection.

### Implementation of generative embedding

#### First-level analysis

The first level of our statistical analysis employed a mass-univariate analysis in each subject. Each auditory stimulus was modelled as a separate delta function, and the resulting trains of auditory events were convolved with a canonical haemodynamic response function. The first regressor in the design matrix contained all auditory events (i.e., normal and time-reversed speech stimuli); the second regressor modelled intelligibility (normal vs. time-reversed speech) as a parametric modulation. Beta coefficients were estimated for all brain voxels using the general linear model [1]. To identify regions responding to auditory stimulation per se, we used an ‘all auditory events’ contrast based on the first regressor (i.e., a contrast between auditory stimuli and background scanner noise), designed to find early auditory regions required for the perception of any broad-band stimulus, whether it is speech or speech-like.

#### Second-level (group) analysis

**Figure 3:**
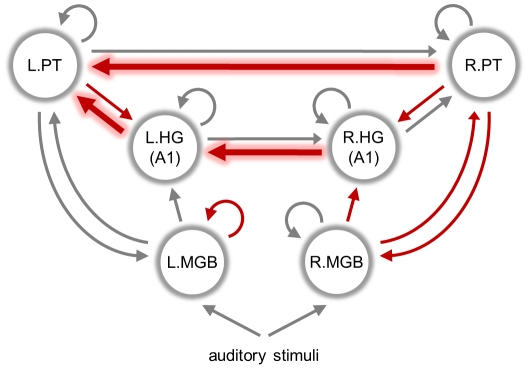
Dynamic causal model of speech processing. The diagram illustrates the specific dynamic causal model (DCM) that was used for the illustrative application of generative embedding in this study. It consists of 6 regions (circles), 15 interregional connections (straight arrows between regions), 6 self-connections (circular arrows), and 2 stimulus inputs (straight arrows at the bottom). The specific set of connections shown here is the result of Bayesian model selection that was carried out on the basis of a large set of competing connectivity layouts (for details, see Schofield et al., *in preparation*). A sparse set of 9 out of 23 connectivity and input parameters (see Figure 10) was found to be sufficiently informative to distinguish between aphasic patients and healthy controls with near-perfect accuracy (see Figure 5). The connections corresponding to these 9 parameters are highlighted in red. Only three parameters were selected in all cross-validation folds and are thus particularly meaningful for classification (bold red arrows); these refer to connections mediating information transfer from the right to the left hemisphere, converging on left PT, which is a key structure in speech processing.

The second level analysis served to select regions whose voxels entered the subject-specific DCMs (in terms of the first eigenvariate of their time series). In the previous study of these data (Schofield et al., *in preparation*), we had compared a set of 512 alternative DCMs that embodied competing hypotheses about the architecture of the thalamo-temporal network processing speech-like stimuli per se. Here, we focus on the model which was found to have the highest evidence in our previous study, i.e., the model providing the best trade-off between accuracy and complexity in explaining the data [94], [83], [88]. Note that this selection procedure is ignorant of subject labels, which prevents test labels from influencing the training procedure. (An alternative, computationally more expensive approach would be to select the model that affords the best classification accuracy, and integrate this selection step into an overall cross-validation scheme. See [61] for an example.) In addition, the selection of time series remains independent of the test data. The DCM we used contains 6 regions (medial geniculate body, MGB; Heschl's gyrus, HG; planum temporale, PT), three in each hemisphere, and 14 interregional connections (see Figure 3). Note that this model concerned processing of acoustic stimuli with speech-like spectral properties *per se*, not differentiating between normal and time-reversed speech; therefore, it did not contain modulatory inputs (corresponding to an empty 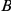 matrix, see Eqn. 4). Critically, instead of identifying regions functionally by a group contrast, we pre-defined large anatomical masks (16 mm×16 mm×16 mm) that specified only the rough location of the 6 regions of interest (see Table 1 and Supplementary Material). These masks served to guide the selection of time series, using a leave-one-out approach to feature selection as described below.

**Table 1:** Regions of interest.

Region		MNI coordinates
L.MGB	left medial geniculate body	−23 mm, −23 mm, −1 mm
L.HG	left Heschl's gyrus (A1)	−47 mm, −26 mm, 7 mm
L.PT	left planum temporale	−64 mm, −23 mm, 8 mm
R.MGB	right medial geniculate body	22 mm, −21 mm, −1 mm
R.HG	right Heschl's gyrus (A1)	48 mm, −24 mm, 6 mm
R.PT	right planum temporale	65 mm, −22 mm, 3 mm

Speech processing can be modelled using a dynamic causal model (DCM) with 6 regions. The table lists the central coordinates of these regions in MNI152 space. These coordinates define the centre of the rough anatomical masks (16 mm×16 mm×16 mm) that guided the specification of the exact location and extent of the regions of interest underlying model inversion (see Section ‘Implementation of generative embedding’). For an illustration of these masks, see Figure S1 in the Supplementary Material.

#### Model specification

To specify the exact location and extent of our 6 regions of interest, and thus the exact time series that would be modelled by DCM, we used a leave-one-out approach to feature selection. For this purpose, we carried out 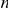 separate second-level analyses, each time leaving out one subject, and then used a conventional summary-statistic approach [95] across the remaining 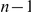 subjects to find voxels that survived a one-sample ‘all auditory events’ t-test with a statistical threshold of 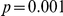 (uncorrected), across all subjects, within the anatomical masks described above. Note that this contrast is agnostic about diagnostic status (corresponding to Figure 2c). (With the cross-validation scheme used here, a between-group contrast could have been used as well without risking bias; see Section ‘Strategies for unbiased model specification and inversion’. This case would correspond to Figure 2f.) Within each leave-one-out repetition, our procedure yielded 6 voxel sets, one for each region of interest. We used the first eigenvariate over voxels as a representative time series for each region in DCM.

#### Model inversion

Inversion of the DCM was carried out independently for each subject, and separately for each cross-validation fold (i.e., each group contrast). With regions (and thus modelled time series) differing each time depending on the current set of 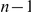 subjects, this procedure resulted in a total of 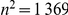 fitted DCMs. We emphasize once more that model inversion was carried out in an unsupervised fashion, i.e., without reference to the subjects' diagnostic status.

#### Kernel construction

A generative score space was constructed on the basis of the MAP estimates of the neuronal model parameters (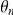 in Eqn. 5). The rzzesulting space contained 22 features: 20 interregional connection strengths (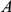 matrix), no modulatory parameters (as the 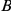 matrix was empty in the DCM we used), and 2 input parameters (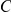 matrix). All feature vectors were normalized to unit length. To minimize the risk of overfitting and enable a clear interpretation of feature weights, we used a linear kernel. Consequently, the similarity between two subjects was defined as the inner product between the normalized vectors of the posterior means of their model parameters.

#### Classification

**Figure 4:**
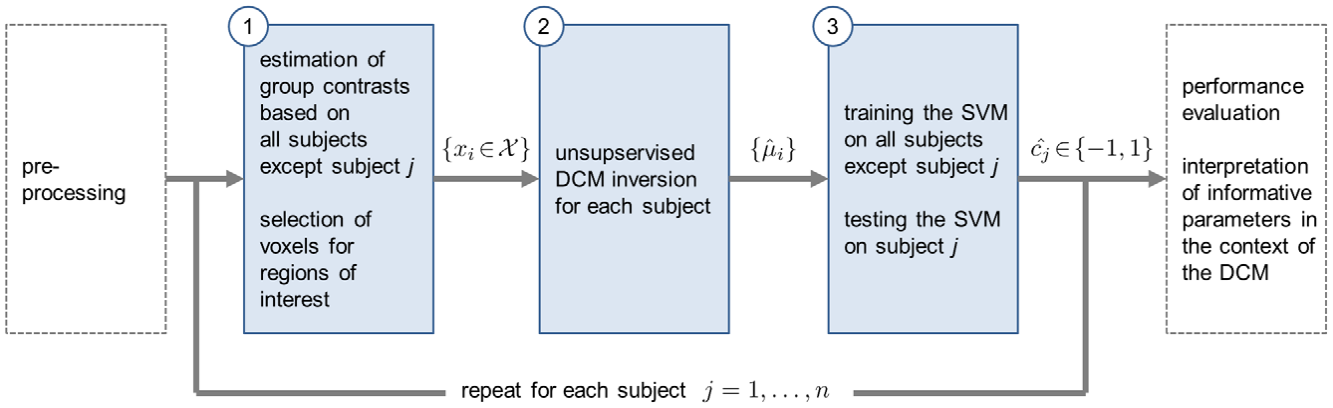
Practical implementation of generative embedding for fMRI. This figure summarizes the three core steps involved in the practical implementation of generative embedding proposed in this paper. This procedure integrates the inversion of a generative model into cross-validation. In step 1, within a given repetition 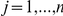, the model is specified using all subjects except 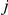. This yields a set of time series 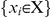 for each subject 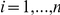. In step 2, the model is inverted independently for each subject, giving rise to a set of subject-specific posterior parameter means 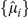. In step 3, these parameter estimates are used to train a classifier on all subjects except 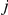 and test it on subject 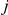, which yields a prediction about the class label of subject 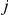. After having repeated these three steps for all 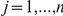, the set of predicted labels can be compared with the true labels, which allows us to estimate the algorithm's generalization performance. In addition, parameters that proved jointly discriminative can be interpreted in the context of the underlying generative model. The sequence of steps shown here corresponds to the procedure shown in Figure 2c and 2f, where it is contrasted with alternative procedures that are simpler but risk an optimistic bias in estimating generalization performance.

An 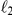-norm soft-margin linear support vector machine (SVM) was trained and tested using leave-one-out cross-validation. Specifically, in each fold 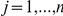, the classifier was trained on all subjects except 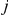, on the basis of the DCM parameter estimates obtained from fitting the voxel time series selected by the group analysis based on all subjects except 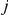. The classifier was then tested by applying it to DCM parameter estimates for the time series from subject 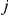 (using the same voxels as the rest of the group). Crucially, in this way, test data and test labels were neither used for model specification nor for classifier training, preventing optimistic estimates of classification performance. The principles of this unbiased procedure are summarized in Figure 4.

Within each fold, the complexity penalty 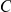 of the SVM was selected by a line search in log_2_ space, to minimize an estimate of the generalization error on the training set (nested cross-validation). To discourage the classifier from acquiring a bias in favour of the majority class, the training set was balanced using a stochastic oversampling strategy. We assessed the generalization performance of the classifier by comparing its 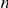 predictions to the 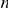 true subject labels (‘patient’ or ‘healthy control’), resulting in a 2×2 confusion matrix that forms the basis of various common performance measures, such as the accuracy or the area under the receiver-operator characteristic (ROC) curve.

### Comparative analyses

We compared the performance of generative embedding to a range of alternative approaches. To begin with, we examined several conventional activation-based classification schemes. The first method was based on a feature space composed of all voxels within the predefined anatomical masks used for guiding the specification of the DCMs. As above, we used a linear SVM, and all training sets were balanced by oversampling. We will refer to this approach as *anatomical feature selection*.

The second method, in contrast to the first one, was not only based on the same classifier as in generative embedding but also used exactly the same voxels. Specifically, voxels were selected on the basis of the same ‘all auditory events’ contrast as above, which is a common approach to defining a voxel-based feature space in subject-by-subject classification [11], [12], [10]. In every cross-validation fold, only those voxels entered the classifier that survived a t-test (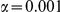, uncorrected) in the current set of 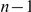 subjects. Training sets were balanced by oversampling. We will refer to this method as *contrast feature selection*.

The third activation-based method employed a locally multivariate ‘searchlight’ strategy for feature selection. Specifically, in each cross-validation fold, a searchlight sphere (radius 4 mm) was passed across all voxels contained in the anatomical masks described above [23]. Using the training set only, a nested leave-one-out cross-validation scheme was used to estimate the generalization performance of each sphere using a linear SVM with a fixed regularization hyperparameter (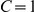). Next, all spheres with an accuracy greater than 75% were used to form the feature space for the current outer cross-validation fold, which corresponds to selecting all voxels whose local neighbourhoods allowed for a significant discrimination between patients and healthy controls at 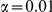. Both outer and inner training sets were balanced by oversampling. We will refer to this method as *searchlight feature selection*. To illustrate the location of the most informative voxels, we carried out an additional searchlight analysis, based on the entire dataset as opposed to a subset of size 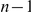, and used the results to generate a discriminative map (see Figure S1 in the Supplementary Material).

The fourth conventional method was based on a principal component analysis (PCA) to reduce the dimensionality of the feature space constructed from all voxels in the anatomical masks described above. Unlike generative embedding, PCA-based dimensionality reduction finds a linear manifold in the data without a mechanistic view of how those data might have been generated. We sorted all principal components in decreasing order of explained variance. By retaining the 22 top components, the resulting dimensionality matched the dimensionality of the feature space used in generative embedding.

In addition to the above activation-based methods, we compared generative embedding to several approaches based on undirected regional correlations. We began by averaging the activity within each region of interest to obtain region-specific representative time series. We then computed pairwise correlation coefficients to obtain a 15-dimensional feature space of functional connectivity. Next, instead of computing spatial averages, we summarized the activity within each region in terms of the first eigenvariate. Thus, in this approach, the exact same data was used to estimate functional connectivity as was used by DCM to infer effective connectivity. Finally, as suggested in [43], we created yet another feature space by transforming the correlation coefficients on eigenvariates into z-scores using the Fisher transformation [96].

**Figure 5:**
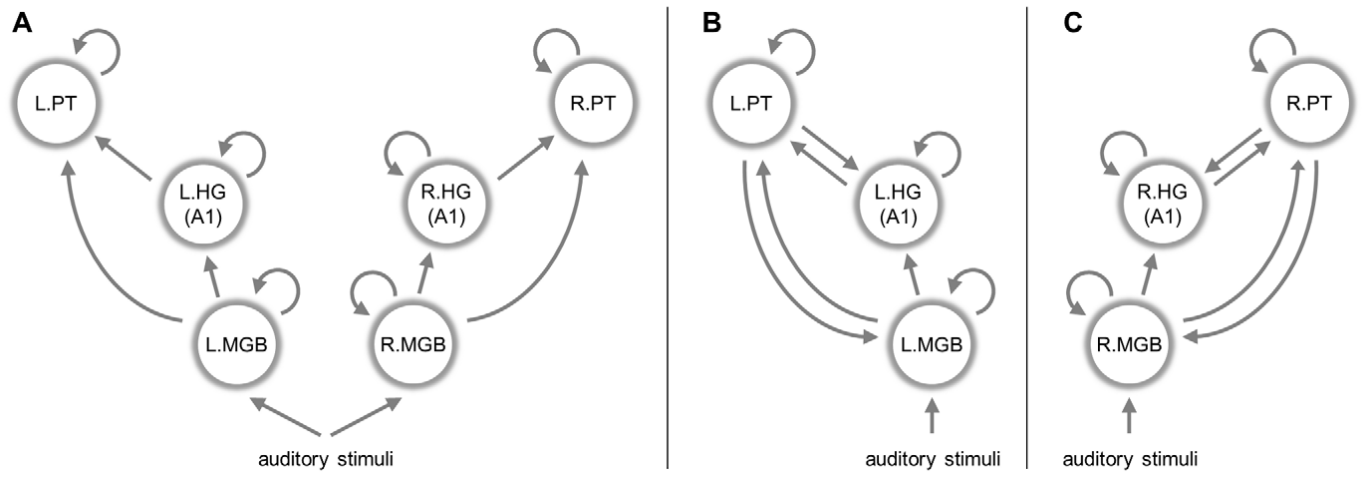
Biologically unlikely alternative models. To illustrate the specificity of generative embedding, the analysis described in the main text was repeated on the basis of three biologically less plausible models. In contrast to the full model shown in Figure 3, these alternative models either (a) contained no feedback or interhemispheric connections, (b) accounted for activity in the left hemisphere only, or (c) focussed exclusively on the right hemisphere. For results, see Table 2 and Figure 6.

In addition to conventional activation- and correlation-based approaches, we also investigated the dependence of generative embedding on the structure of the underlying model. Specifically, we repeated our original analysis on the basis of three alternative models. For the first model, we constructed a *feedforward* system by depriving the original model of all feedback and interhemispheric connections (Figure 5a); while this model could still, in principle, explain neuronal dynamics throughout the system of interest, it was neurobiologically less plausible. For the second and third model, we kept all connections from the original model but modelled either only the *left hemisphere* (Figure 5b) or only the *right hemisphere* (Figure 5c).

In summary, we compared the primary approach proposed in this paper to 4 conventional activation-based methods, 3 conventional correlation-based methods, and 3 generative-embedding analyses using reduced and biologically less plausible models.

## Results

### Classification performance

**Figure 6:**
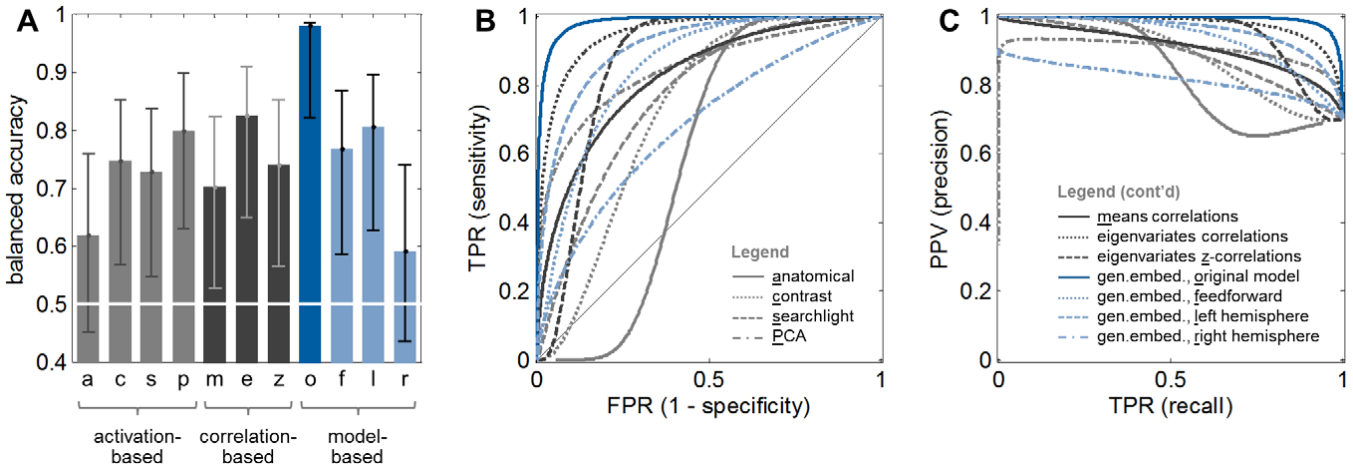
Classification performance. Classification based on generative embedding using the model shown in Figure 3 was compared to ten alternative methods: anatomical feature selection, contrast feature selection, searchlight feature selection, PCA-based dimensionality reduction, regional correlations based on region means, regional correlations based on eigenvariates, regional z-transformed correlations based on eigenvariates, as well as generative embedding using three biologically unlikely alternative models (see inset legends for abbreviations). (a) The balanced accuracy and its central 95% posterior probability interval show that all methods performed significantly better than chance (50%) with the exception of classification with anatomical feature selection and generative embedding using a nonsensical model. Differences between activation-based methods (light grey) and correlation-based methods (dark grey) were largely statistically indistinguishable. By contrast, using the full model shown in Figure 3, generative embedding (blue) significantly outperformed all other methods, except when used with biologically unlikely models (Figure 5). (b) Receiver-operating characteristic (ROC) curves of the eleven methods illustrate the trade-off between true positive rate (sensitivity) and false positive rate (1 – specificity) across the entire range of detection thresholds. A larger area under the curve is better. (c) Precision-recall (PR) curves illustrate the trade-off between positive prediction value (precision) and true positive rate (recall). A larger area under the curve is better. Smooth ROC and PR curves were obtained using a binormal assumption on the underlying decision values [97]. For a numerical summary of all results, see Table 2.

The classification performance of generative embedding was evaluated using the procedure described in Figure 2c. This procedure was compared to several conventional activation-based and correlation-based approaches. As an additional control, generative embedding was carried out on the basis of three biologically ill-informed models. In all cases, a leave-one-subject-out cross-validation scheme was used to obtain the posterior distribution of the balanced accuracy [92] as well as smooth estimates of the underlying receiver-operating characteristic (ROC) and precision-recall (PC) curves [97]. Results are presented in Table 2 and Figure 6.

**Table 2:** Classification results.

Measure (*n* = 37)	Anatomical feature selection	Contrast feature selection	Searchlight feature selection	PCA-based dimensionality reduction
(1) Accuracy	0.649	0.757	0.730	0.865
(2) Balanced accuracy	0.619	0.748	0.729	0.799
(3) Significantly above chance	*p*≈0.089	*p*≈0.003	*p*≈0.006	*p*<0.001
(4) True positive rate (TPR; sensitivity; recall)	0.545	0.727	0.727	0.636
(5) True negative rate (TNR; specificity)	0.692	0.769	0.731	0.962
(6) Positive predictive value (PPV; precision)	0.429	0.571	0.533	0.875
(7) Negative predictive value (NPV)	0.783	0.870	0.864	0.862
(8) Area under the ROC curve (AUC)	0.657	0.829	0.794	0.846
(9) Area under the PR curve (average precision)	0.756	0.854	0.842	0.885
**…**	**Region-means correlations**	**Eigenvariates correlations**	**Eigenvariates z-correlations**	
	0.730	0.865	0.784	
	0.703	0.825	0.741	
	*p*≈0.011	*p*<0.001	*p*≈0.002	
	0.636	0.727	0.636	
	0.769	0.923	0.846	
	0.538	0.800	0.636	
	0.833	0.889	0.846	
	0.804	0.958	0.857	
	0.873	0.945	0.914	
**…**	**Generative embedding (full model)**	**Generative embedding (feedforward model)**	**Generative embedding (left hemisphere)**	**Generative embedding (right hemisphere)**
	0.973	0.784	0.838	0.649
	0.981	0.767	0.806	0.593
	*p*<0.001	*p*≈0.001	*p*<0.001	*p*≈0.134
	1.000	0.727	0.727	0.455
	0.962	0.808	0.885	0.731
	0.917	0.615	0.727	0.417
	1.000	0.875	0.885	0.760
	0.990	0.867	0.923	0.706
	0.957	0.916	0.934	0.803

This table contrasts the classification results obtained through generative embedding with those afforded by three conventional methods. As described in the main text, the underlying dataset serves illustrative purposes, and so, due to its small sample size (*n* = 37), all numbers are associated with considerable uncertainty. The measure of primary interest is the balanced accuracy (2). Its uncertainty can be captured by computing a posterior probability interval (as shown in Figure 6a), or by computing a *p*-value (3), which represents the probability with which the observed performance would have been obtained by chance.

The strongest classification performance was obtained when using generative embedding with the full model shown in Figure 3. The approach correctly associated 36 out of 37 subjects with their true disease state, corresponding to a balanced accuracy of 98%. Regarding conventional activation-based methods, classification based on anatomical feature selection did not perform significantly above chance (balanced accuracy 62%, *p*≈0.089). Contrast feature selection (75%, *p*≈0.003), searchlight feature selection (73%, *p*≈0.006), and PCA-based dimensionality reduction (80%, *p*<0.001) did perform significantly above chance; however, all methods were outperformed significantly by generative embedding (*p*≈0.003, *p*≈0.001, and *p*≈0.045, paired-sample Wald test). Regarding conventional correlation-based methods, all three approaches performed above chance, whether based on correlations amongst the means (70%, *p*≈0.011), correlations amongst eigenvariates (83%, *p*<0.001), or z-transformed correlations amongst eigenvariates (74%, *p*≈0.002). Critically, however, all were significantly outperformed by generative embedding (*p*<0.001, *p*≈0.045, *p*≈0.006). Regarding generative embedding itself, when replacing the original model shown in Figure 3 by a biologically less plausible feedforward model (Figure 5a) or by a model that captured the left hemisphere only (Figure 5b), we observed a significant decrease in performance, from 98% down to 77% (*p*≈0.002) and 81% (*p*≈0.008), respectively, although both accuracies remained significantly above chance (*p*≈0.001 and *p*<0.001). By contrast, when modelling the right hemisphere only (Figure 5c), performance dropped to a level indistinguishable from chance (59.3%, *p*≈0.134).

**Figure 7:**
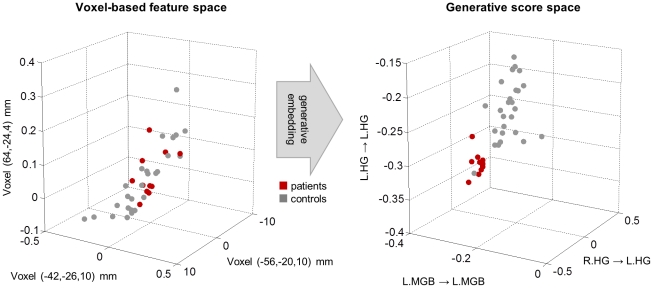
Induction of a generative score space. This figure provides an intuition of how a generative model transforms the data from a voxel-based feature space into a generative score space (or model-based feature space), in which classes become more separable. The left plot shows how aphasic patients (red) and healthy controls (grey) are represented in voxel space, based on t-scores from a simple ‘all auditory events’ contrast (see main text). The three axes represent the peaks of those three clusters that showed the strongest discriminability between patients and controls, based on a locally multivariate searchlight classification analysis. They are located in L.PT, L.HG, and R.PT, respectively (cf. Table 1). The right plot shows the three individually most discriminative parameters (two-sample t-test) in the (normalized) generative score space induced by a dynamic causal model of speech processing (see Figure 3). The plot illustrates how aphasic patients and healthy controls become almost perfectly linearly separable in the new space. Note that this figure is based on normalized examples (as used by the classifier), which means the marginal densities are not the same as those shown in Figure 9 but are exactly those seen by the classifier. A stereogram of the generative score space can be found in the Supplementary Material (Figure S4).

In order to provide a better intuition as to how the generative model shown in Figure 3 created a score space in which examples were much better separated than in the original voxel-based feature space, we produced two scatter plots of the data (see Figure 7). The first plot is based on the peak voxels of the three most discriminative clusters among all regions of interest, evaluated by a searchlight classification analysis. The second plot, by analogy, is based on the three most discriminative model parameters, as measured by two-sample t-tests in the (normalized) generative score space. This illustration shows how the voxel-based projection (left) leads to classes that still overlap considerably, whereas the model-based projection (right) provides an almost perfectly linear separation of patients and controls.

### Characterization of the feature space

**Figure 8:**
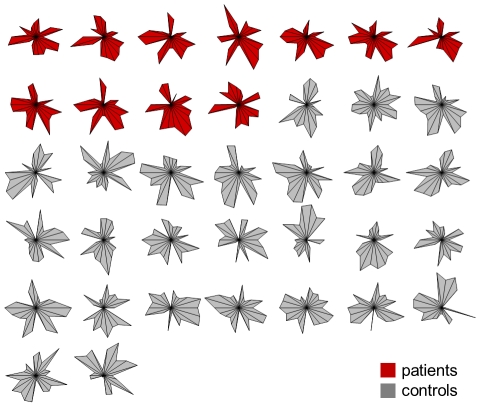
Connectional fingerprints. Given the low dimensionality of the model-induced feature space, subjects can be visualized in terms of ‘connectional fingerprints’ [98] that are based on a simple radial coordinate system in which each axis corresponds to the *maximum a posteriori* (MAP) estimate of a particular model parameter. The plot shows that the difference between aphasic patients (red) and healthy controls (grey) is not immediately obvious, suggesting that it might be subtle and potentially of a distributed nature.

The low dimensionality of the model-based feature space makes it possible to visualize each example in a radial coordinate system, where each axis corresponds to a particular model parameter (see Figure 8). When using parameters that represent directed connection strengths, this form of visualization is reminiscent of the notion of ‘connectional fingerprints’ for characterizing individual cortical regions [98]. In our case, there is no immediately obvious visual difference in fingerprints between aphasic patients and healthy controls. On the contrary, the plot gives an impression of the large variability across subjects and suggests that differences might be subtle and possibly jointly encoded in multiple parameters.

**Figure 9:**
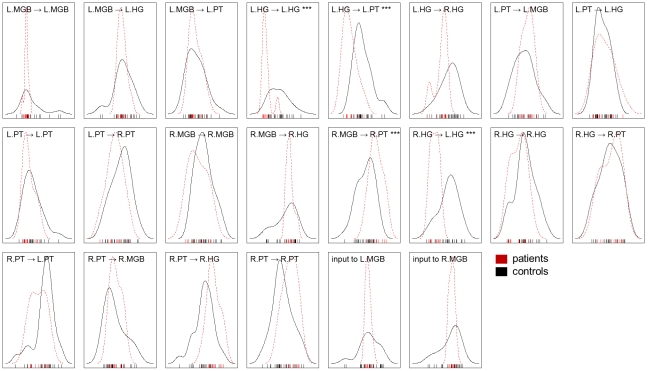
Univariate feature densities. Separately for patients (red) and healthy controls (grey), the figure shows nonparametric estimates of the class-conditional densities of the *maximum a posteriori* (MAP) estimates of model parameters. The estimates themselves are shown as a rug along the x-axis. The results of individual (uncorrected) two-sample t-tests, thresholded at *p* = 0.05, are indicated in the title of each diagram. Three stars (***) correspond to *p*<0.001, indicating that the associated model parameter assumes very different values for patients and controls.

One way of characterizing the discriminative information encoded in individual model parameters more directly is to estimate class-conditional univariate feature densities (see Figure 9). Here, densities were estimated in a nonparametric way using a Gaussian kernel with an automatically selected bandwidth, making no assumptions about the distributions other than smoothness [99]. While most densities are heavily overlapping, a two-sample t-test revealed significant group differences in four model parameters (denoted by stars in Figure 9): the self-connection of L.HG (parameter 4); the influence that L.HG exerts over L.PT (parameter 5); the influence R.MGB on R.PT (parameter 13); and the influence of R.HG on L.HG (parameter 14). All of these were significant at the 0.001 level while no other parameter survived *p* = 0.05. An extended plot of all bivariate feature distributions, illustrating how well any two features jointly discriminated between patients and healthy controls, can be found in the Supplementary Material (Figure S2).

**Figure 10:**
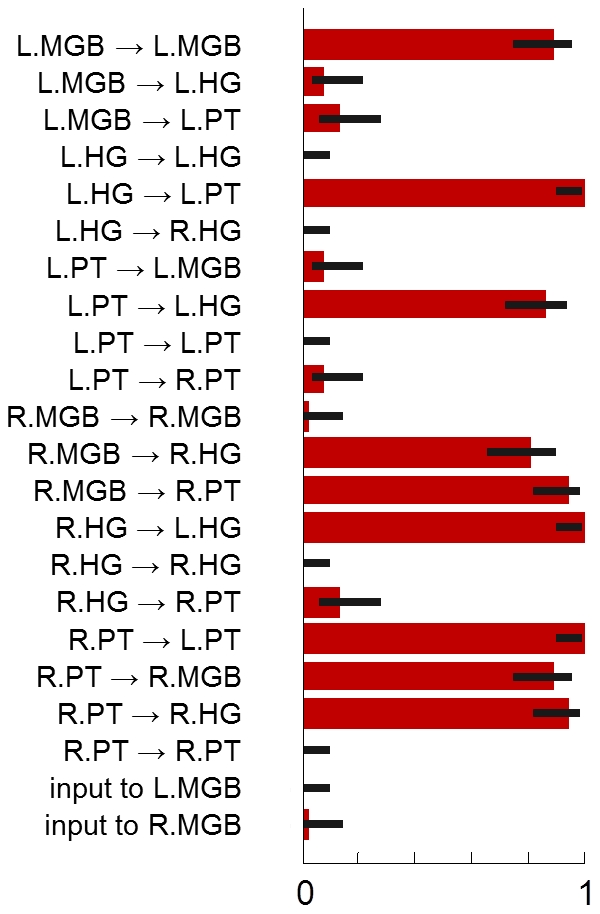
Discriminative features. A support vector machine with a sparsity-inducing regularizer (capped 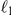-regularizer) was trained and tested in a leave-one-out cross-validation scheme, resulting in 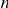 subsets of selected features. The figure summarizes these subsets by visualizing how often each feature (printed along the y-axis) was selected across the 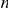 repetitions (given as a fraction on the x-axis). Error bars represent central 95% posterior probability intervals of a Beta distribution with a flat prior over the interval [0, 1]. A group of 9 features was consistently found jointly informative for discriminating between aphasic patients and healthy controls (see main text). An additional figure showing which features were selected in each cross-validation fold can be found in the Supplementary Material (Figure S3). Crucially, since each feature corresponds to a model parameter that describes one particular interregional connection strength, the group of informative features can be directly related back to the underlying dynamic causal model (see highlighted connections in Figure 3).

In order to better understand which DCM parameters jointly enabled the distinction between patients and controls, we examined the frequency with which features were selected in leave-one-out cross-validation when using an SVM with a sparsity-inducing regularizer [75], [74] (see Figure 10). We found that the classifier favoured a highly consistent and sparse set of 9 (out of 22) model parameters; the corresponding synaptic connections are highlighted in red in Figure 3. Notably, this 9-dimensional feature space, when used with the original 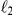-norm SVM, yielded the same balanced classification accuracy (98%) as the full 22-dimensional feature space, despite discarding more than two thirds of its dimensions.

The above representation disclosed interesting potential mechanisms. For example, discriminative parameters were restricted to cortico-cortical and thalamo-cortical connection strengths, whereas parameters representing auditory inputs to thalamic nuclei did not contribute to the distinction between patients and healthy controls. This finding implies that, as one would expect, low-level processing of auditory stimuli, from brain stem to thalamus, is unimpaired in aphasia and that processing deficiencies are restricted to thalamo-cortical and cortico-cortical networks. In particular, the discriminative connections included the top-down connections from planum temporale to Heschl's gyrus bilaterally; the importance of these connections had also been highlighted by the previous univariate analyses of group-wise DCM parameters in the study by Schofield et al. (*in preparation*). Furthermore, all of the connections from the right to the left hemisphere were informative for group membership, but none of the connections in the reverse direction. This pattern is interesting given the known specialization of the left hemisphere in language and speech processing and previous findings that language-relevant information is transferred from the right hemisphere to the left, but not vice versa [100]. It implies that aphasia leads to specific changes in connectivity, even in non-lesioned parts of the language network, with a particular effect on inter-hemispheric transfer of speech information. This specificity is seen even more clearly when considering only those three parameters which were selected 100% of the time (i.e., in all cross-validation folds) and are thus particularly meaningful for classification (bold red arrows in Figure 3). The associated connections mediate information transfer from the right to the left hemisphere and converge on the left planum temporale which is a critical structure for processing of language and speech [101], [102].

In summary, all selected features represented connectivity parameters (as opposed to stimulus input), their selection was both sparse and highly consistent across resampling repetitions, and their combination was sufficient to afford the same classification accuracy as the full feature set.

## Discussion

### Perspectives for generative embedding of fMRI data

Generative embedding for subject-by-subject classification provides three potential advantages over conventional voxel-based methods. The first advantage is that it combines the explanatory strengths of generative models with the classification power of discriminative methods. Thus, in contrast to purely discriminative or purely generative methods, generative embedding is a hybrid approach. It fuses a feature space that captures both the data and their generative process with a classifier that finds the maximum-margin boundary for class separation. Intuitively, this exploits the idea that differences in the generative process between two examples (observations) might provide optimal discriminative information required to enable accurate predictions. In the case of DCM for fMRI, this rationale should pay off whenever the directed connection strengths between brain regions contain more information about a disease state than regional activations or undirected correlations. Indeed, this is what we found in our analyses (cf. Figure 6). Using a DCM-informed data representation might prove particularly relevant in psychiatric disorders, such as schizophrenia or depression, where aberrant effective connectivity and synaptic plasticity are central to the disease process [48], [47].

The second advantage of generative embedding for fMRI is that it enables an intuitive and mechanistic interpretation of features and their weights, an important property not afforded by most conventional classification methods [103], [104]. By using parameter estimates from a mechanistically interpretable model for constructing a feature space, the subsequent classification no longer yields ‘black box’ results but allows one to assess the relative importance of different mechanisms for distinguishing groups (e.g., whether or not synaptic plasticity alters the strengths of certain connections in a particular context). Put differently, generative embedding embodies a shift in perspective: rather than representing sequential data in terms of high-dimensional and potentially highly complex trajectories, we are viewing the data in terms of the coefficients of a well-behaved model of system dynamics. Again, this may be of particular importance for clinical applications, as discussed in more detail below. It is also interesting to note that models like DCM, when used in the context of generative embedding, turn the curse of dimensionality faced by conventional classification methods into a blessing: the higher the spatial and temporal resolution of the underlying fMRI data, the more precise the resulting DCM parameter estimates; this in turn should lead to more accurate predictions.

The third advantage provided by generative embedding is related to model comparison. For any given dataset, there is an infinite number of possible dynamic causal models, differing in the number and location of nodes, in connectivity structure, and in their parameterization (e.g., priors). Competing models can be compared using Bayesian model selection (BMS) [89], [83], [86], [88], where the best model is the one with the highest model evidence, that is, the highest probability of the data given the model [105]. BMS is a generic approach to distinguish between different models that is grounded in Bayesian probability theory and, when group-specific mechanisms can be mapped onto distinct models, represents a powerful technique for model-based classification in itself. However, there are two scenarios in which BMS is problematic and where classification based on generative embedding may represent a useful alternative [61]. First, BMS requires the data to be identical for all competing models. Thus, in the case of current implementations of DCM for fMRI, BMS enables *dynamic* model selection (concerning the parameterization and mathematical form of the model equations) but not *structural* model selection (concerning which regions or nodes should be included in the model). Second, BMS is limited when different groups cannot be mapped onto different model structures, for example when the differences in neuronal mechanisms operate at a finer conceptual scale than can be represented within the chosen modelling framework. In this case, discriminability of subjects may be afforded by differences in (combinations of) parameter estimates under the same model structure (see [106] for a recent example).

In both these scenarios, the approach proposed in this paper may provide a solution, in that the unsupervised creation of a generative score space can be viewed as a method for biologically informed feature extraction, and the performance of the classifier reflects how much class information is encoded in the model parameters. This view enables a form of model comparison in which the best model is the one that enables the highest classification accuracy. This procedure can be applied even when (i) the underlying data (e.g., the chosen regional fMRI time series) are different, or when (ii) the difference between two models lies exclusively in the pattern of parameter estimates. In this paper, we have illustrated both ideas: *structural* model selection to decide between a full model and two reduced models that disregard one hemisphere; and *dynamic* model selection to distinguish between different groups of subjects under the same model structure.

In summary, BMS evaluates the goodness of a model with regard to its generalizability for explaining the data, whereas generative embedding evaluates a model in relation to an external criterion, i.e., how well it allows for inference on group membership of any given subject. This difference is important as it highlights that the concept of a ‘good’ model can be based on fundamentally different aspects, and one could imagine scenarios where BMS and generative embedding arrive at opposing results. If, for example, discriminability of groups relies on a small subspace of the data, then one model (which provides a good accuracy-complexity trade-off for most of the data except that subspace) may have higher evidence, but another model that describes this subspace particularly well but is generally worse for the rest of the data may result in better classification performance (cf. our discussion in [106]). We will examine the relation and complementary nature of BMS and generative-embedding approaches in future work.

As discussed in this paper, there are three valid strategies for the implementation of generative embedding in fMRI that allow for an unbiased estimate of classification accuracy (Figure 2). If regions (and thus time series) are defined anatomically, the model is inverted separately for each subject, and the resulting parameter estimates can be safely used in cross-validation. If regions are defined by a functional contrast, both time series selection and model inversion for all subjects need to be carried out separately for each cross-validation fold. These procedures clearly have higher computational demands than conventional classification techniques, but the subject-wise nature of model inversion means that generative embedding for fMRI can exploit methods for distributed computing and can thus be implemented even for larger numbers of subjects.

### Summary of our findings

In order to demonstrate the utility of generative embedding for fMRI, we acquired and analysed a dataset consisting of 11 aphasic patients and 26 healthy controls. During the experiment, participants were listening to a series of speech and speech-like stimuli. In an initial analysis (Schofield et al., *in preparation*), we designed a dynamic causal model to explain observed activations in 6 auditory regions of interest. Here, we extended this analysis by examining whether patients and healthy controls could be distinguished on the basis of differences in subject-specific generative models. Specifically, we trained and tested a linear support vector machine on subject-wise estimates of connection strengths. This approach delivered two sets of results.

First, we found strong evidence in favour of the hypothesis that aphasic patients and healthy controls may be distinguished on the basis of differences in the parameters of a generative model alone. Generative embedding did not only yield a near-perfect balanced classification accuracy (98%). It also significantly outperformed conventional activation-based methods, whether they were based on anatomical (62%), contrast (75%), searchlight feature selection (73%), or on a PCA-based dimensionality reduction (80%). Similarly, our approach outperformed conventional correlation-based methods, whether they were based on regional means (70%) or regional eigenvariates (83% and 74%). Furthermore, it is interesting to observe that group separability was reduced considerably when using a less plausible feedforward model (77%). Finally, performance decreased significantly when modelling only the left hemisphere (81%), and it dropped to chance when considering the right hemisphere by itself (60%), which is precisely what one would expect under the view that the left hemisphere is predominantly, but not exclusively, implicated in language processing. Taken together, our findings provide strong support for the central idea of this paper: that critical differences between groups of subjects may be expressed in a highly nonlinear manifold which remains inaccessible by methods relying on activations or undirected correlations, but which can be unlocked by the nonlinear transformation embodied by an appropriate generative model.

Second, since features correspond to model parameters, our approach allowed us to characterize a subset of features (Figure 10) that can be interpreted in the context of the underlying model (Figure 3). This subset showed four remarkable properties. (i) Discriminative parameters were restricted to cortico-cortical and thalamo-cortical connection strengths. On the contrary, parameters representing auditory inputs to thalamic nuclei did not contribute to the distinction between patients and healthy controls. (ii) We observed a high degree of stability across resampling folds. That is, the same 9 (out of 22) features were selected on almost every repetition. (iii) The set of discriminative parameters was found to be sparse, not just within repetitions (which is enforced by the underlying regularizer) but also across repetitions (which is not enforced by the regularizer; see Figure S3 in the Supplementary Material). At the same time, the set was considerably larger than what would be expected from univariate feature-wise t-tests (Figure 9). (iv) The sparse set of discriminative parameters proved sufficient to yield the same balanced classification accuracy (98%) as the full set. These results are consistent with the notion that a distinct mechanism, and thus few parameters, are sufficient to explain differences in processing of speech and speech-like sounds between aphasic patients and healthy controls. In particular, all of the connections from the right to the left hemisphere were informative with regard to group membership, but none of the connections in the reverse direction. This asymmetry resonates with previous findings that language-relevant information is transferred from the right hemisphere to the left, but not vice versa [100], and suggests that in aphasia connectivity changes in non-lesioned parts of the language network have particularly pronounced effects on inter-hemispheric transfer of speech information from the (non-dominant) right hemisphere to the (dominant) left hemisphere.

It is worthwhile briefly commenting on how the present findings relate to those of the original DCM study by Schofield et al. (*in preparation*). Two crucial differences are that the previous study (i) applied Bayesian model averaging to a set of 512 models and (ii) statistically examined each of the resulting average connection strengths in a univariate fashion. They found group differences for most connections, highlighting in particular the top-down connections from planum temporale to primary auditory cortex bilaterally. In our multivariate analysis, these two connections were also amongst the most informative ones for distinguishing patients from controls (Figure 3). Schofield et al. also found group differences for interhemispheric connection strengths between left and right Heschl's gyrus, but their univariate approach did not demonstrate any asymmetries. In contrast, our multivariate approach yielded a sparser set of discriminative connections, highlighting the asymmetries of interhemispheric connections described above (Figure 3).

### Inference on mechanisms for clinical applications

The example described in this paper was chosen to illustrate the implementation and use of generative embedding for fMRI. It is important to emphasize that this example does not represent the sort of clinical application that we envisage in the long term. Clearly, there are few diagnostic problems when dealing with aphasia and usually a clinical examination by the physician is sufficient. However, this example is useful for demonstrating the utility of generative embedding since the diagnostic status of each subject is known without doubt and the networks involved in speech processing are well characterized. In the future, we hope that our approach will be useful for addressing clinical problems of high practical relevance, for instance for dissecting psychiatric spectrum disorders, such as schizophrenia, into physiologically defined subgroups [47], or for predicting the response of individual patients to specific drugs. While an increasing number of studies have tried to describe neurobiological markers for psychiatric disorders [22], [107], [108], [3], [109], [110], [14], [15], we argue that these studies should be complemented by model-based approaches for inferring biologically plausible mechanisms. Such approaches will be useful in two domains of application: they can be used to decide between competing hypotheses (as in traditional applications of DCM and BMS); and they can harvest the potentially rich discriminative information encoded in aspects of synaptic plasticity or neuromodulation to build classifiers that distinguish between different subtypes of a psychiatric disorder on a physiological basis (using techniques such as generative embedding).

In the case of the illustrative dataset analysed in this paper, generative embedding yielded stronger classification performance than conventional methods, whether they were based on activations or regional correlations. One might think that this superior ability to accurately classify individual subjects determines the clinical value of the approach. Instead, we wish to argue that its clinical value will ultimately depend on whether patients that share the same symptoms can be differentially treated according to the underlying pathophysiology of the disorder. Generative embedding, using biologically plausible and mechanistically interpretable models, may prove critical in establishing diagnostic classification schemes that distinguish between pathophysiologically distinct subtypes of spectrum diseases and allow for predicting individualized behavioural and pharmacological therapy.

## Supporting Information

Figure S1
**Further characterization of the voxel-based feature space.** **(**1) Regions of interest. In order to illustrate generative embedding for fMRI, a dynamic causal model was constructed on the basis of 6 anatomical regions of interest. As described in the paper, the exact location of these regions was determined on the basis of an 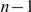 group contrast and hence varied between cross-validation folds. Regions were defined by 16 mm×16 mm×16 mm cubes centred on the group maxima (see Table 1 in the paper). The figure shows the location and extent of the anatomical masks (green) that were used to define fold-specific DCM regions. (2) Searchlight map. A conventional searchlight analysis [23] was carried out to illustrate the degree to which a given voxel and its local spherical environment (radius 4 mm) allowed for a separation between aphasic patients and healthy controls. The map is thresholded at *p* = 0.05 uncorrected and provides a qualitative account of which regions were most informative.(TIF)

Figure S2 **Further characterization of the generative score space.** By analogy with the univariate feature densities shown in Figure 9, the discriminative information encoded in simple combinations of model parameters can be illustrated using bivariate scatter plots. The figure indicates how well any two features jointly discriminated between patients and healthy controls. Note that the matrix is symmetric.
(TIF)

Figure S3
**Feature selection using a sparse SVM.** A support vector machine with a sparsity-inducing regularizer [75] was used to investigate, based on leave-one-out cross validation, which features were typically selected across the underlying 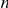 folds. (a) The left figure shows in detail which features were selected in each repetition. For example, when based on all subjects but the first, the classifier selected exactly those 9 features that were selected most of the time; when based on all subjects but the last, a slightly different group of 10 features was favoured. The figure shows that the set of selected features is both sparse and highly consistent across resampling repetitions. As described in the paper, it afforded the same classification accuracy as the full set. (b) The right figure shows the posterior variance of each model parameter, separately for selected and discarded parameters. The data provide no evidence that the algorithm simply selected those parameters that were easier to fit, as would be indicated by a lower posterior variance (two-tailed t-test, *p*≈0.640).(TIF)

Figure S4 **Stereogram of the generative score space.** Based on the generative score space illustrated in the paper (see right plot in Figure 7), we here show the same plot from two slightly different angles. Readers are invited to try and focus an imaginary point behind the two plots, or use a stereoscope, to recover a fully three‐dimensional impression.
(TIF)
