# Supplementary figures and images for: Generative Embedding for Model-Based Classification of fMRI Data
Source: PLoS Comput Biol. 2011 Jun 23;7(6):e1002079. doi: 10.1371/journal.pcbi.1002079 (PMC3121683; doi:10.1371/journal.pcbi.1002079)

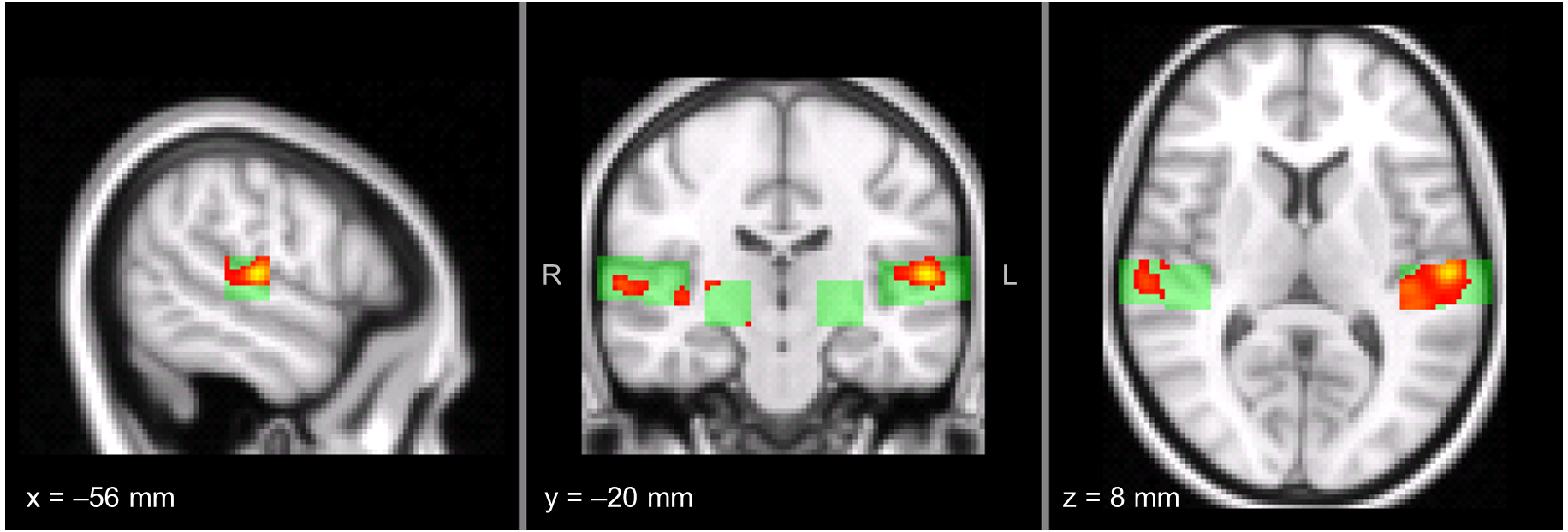

Supplement: Figure S1 — Further characterization of the voxel-based feature space. (1) Regions of interest. In order to illustrate generative embedding for fMRI, a dynamic causal model was constructed on the basis of 6 anatomical regions of interest. As described in the paper, the exact location of these regions was determined on the basis of an group contrast and hence varied between cross-validation folds. Regions were defined by 16 mm×16 mm×16 mm cubes centred on the group maxima (see Table 1 in the paper). The figure shows the location and extent of the anatomical masks (green) that were used to define fold-specific DCM regions. (2) Searchlight map. A conventional searchlight analysis [23] was carried out to illustrate the degree to which a given voxel and its local spherical environment (radius 4 mm) allowed for a separation between aphasic patients and healthy controls. The map is thresholded at p = 0.05 uncorrected and provides a qualitative account of which regions were most informative. (TIF) [file pcbi.1002079.s001.tif]

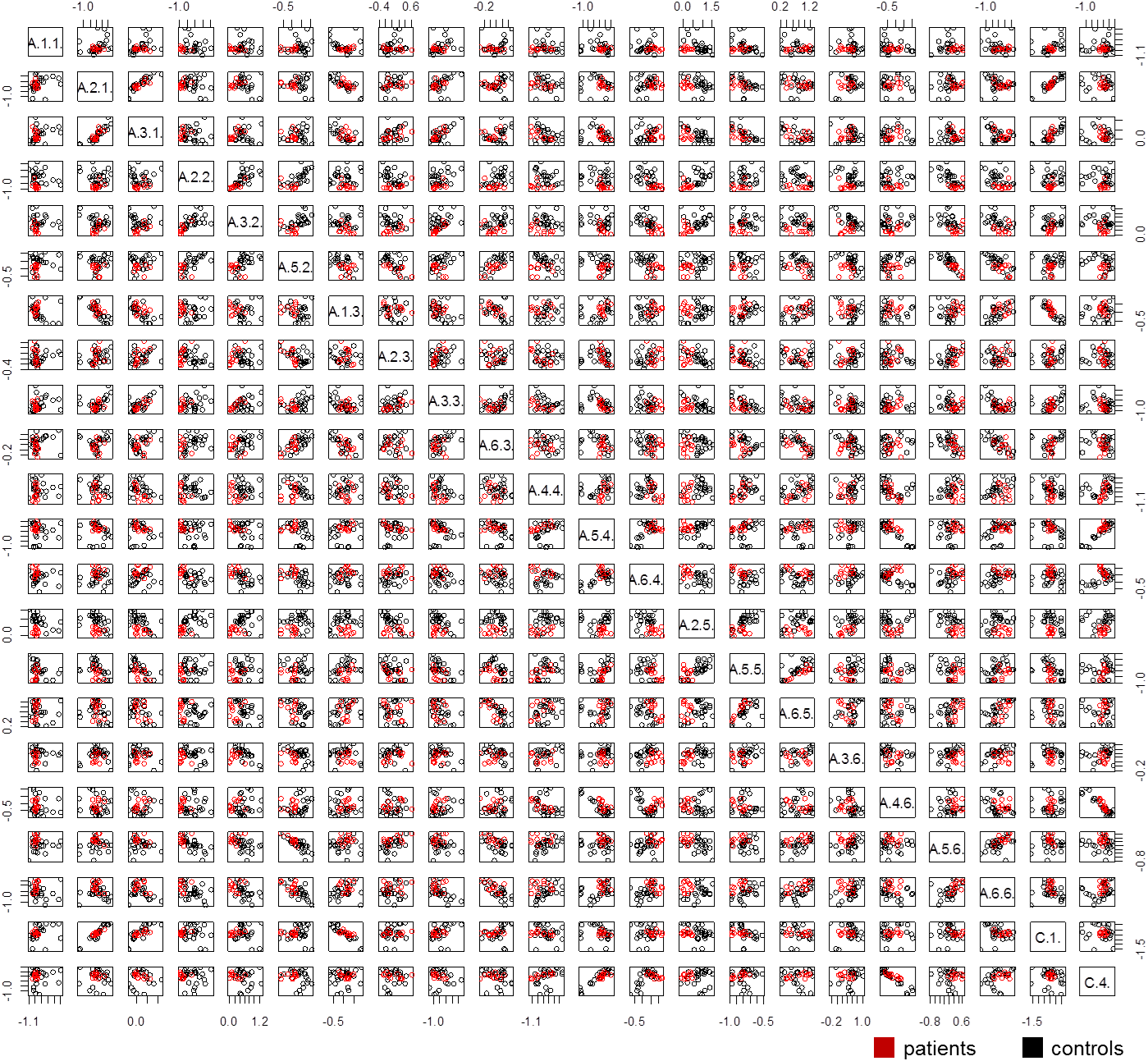

Supplement: Figure S2 — Further characterization of the generative score space. By analogy with the univariate feature densities shown in Figure 9, the discriminative information encoded in simple combinations of model parameters can be illustrated using bivariate scatter plots. The figure indicates how well any two features jointly discriminated between patients and healthy controls. Note that the matrix is symmetric. (TIF) [file pcbi.1002079.s002.tif]

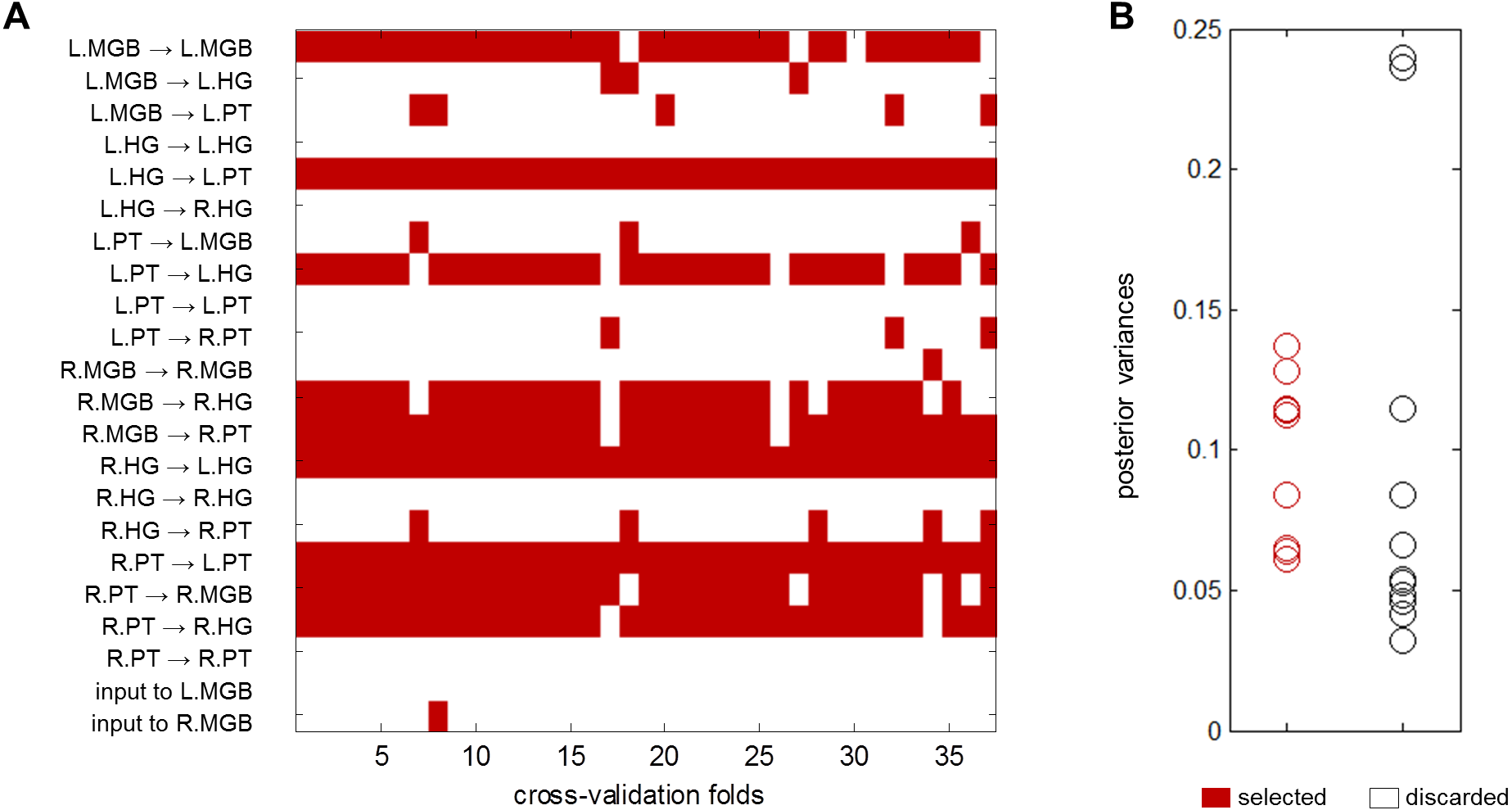

Supplement: Figure S3 — Feature selection using a sparse SVM. A support vector machine with a sparsity-inducing regularizer [75] was used to investigate, based on leave-one-out cross validation, which features were typically selected across the underlying folds. (a) The left figure shows in detail which features were selected in each repetition. For example, when based on all subjects but the first, the classifier selected exactly those 9 features that were selected most of the time; when based on all subjects but the last, a slightly different group of 10 features was favoured. The figure shows that the set of selected features is both sparse and highly consistent across resampling repetitions. As described in the paper, it afforded the same classification accuracy as the full set. (b) The right figure shows the posterior variance of each model parameter, separately for selected and discarded parameters. The data provide no evidence that the algorithm simply selected those parameters that were easier to fit, as would be indicated by a lower posterior variance (two-tailed t-test, p≈0.640). (TIF) [file pcbi.1002079.s003.tif]

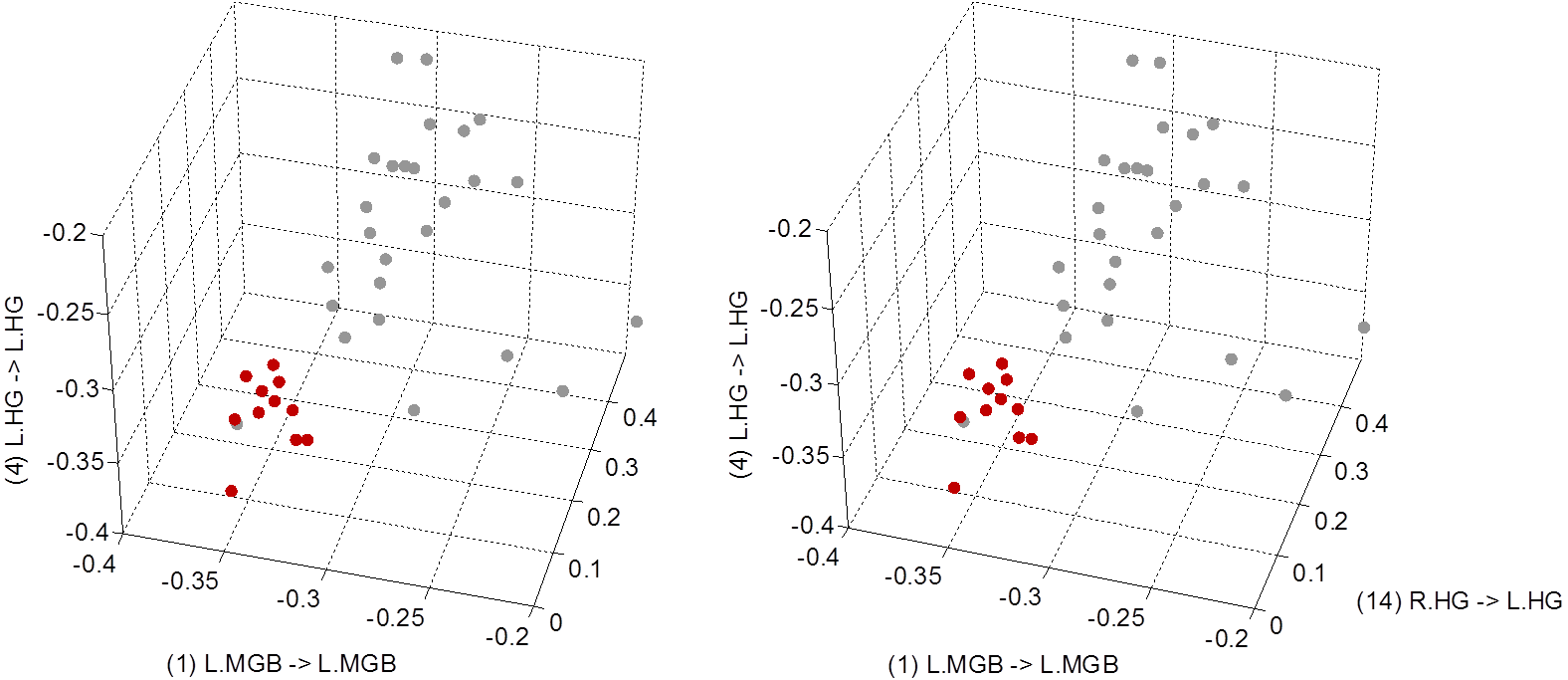

Supplement: Figure S4 — Stereogram of the generative score space. Based on the generative score space illustrated in the paper (see right plot in Figure 7), we here show the same plot from two slightly different angles. Readers are invited to try and focus an imaginary point behind the two plots, or use a stereoscope, to recover a fully three‐dimensional impression. (TIF) [file pcbi.1002079.s004.tif]
